# Efficacy and Safety of Vitex Extract on Menopausal Symptoms and Quality of Life in Postmenopausal Women: A Randomized Placebo-Controlled Trial

**DOI:** 10.7759/cureus.116507

**Published:** 2026-09-18

**Authors:** Prasanna A Reddy, Srilakshmi Aluri, Anzar CA, Joseph MV, Prasad CP, Faizal Mohamed, Shubha M Rani, Hyunjin Lee, Bineesh Earanimose

**Affiliations:** 1 Research and Development, Ingex Botanicals Limited, Bengaluru, IND; 2 Techno Marketing, Ingex Botanicals Limited, Bengaluru, IND; 3 Techno Marketing, Bio Actives Japan Corporation, Tokyo, JPN; 4 Gynecology, Sri Venkateswara Hospital, Bengaluru, IND; 5 Research, Bio Actives Japan Corporation, Tokyo, JPN

**Keywords:** blatt-kupperman index score, hormonal regulation, hot flashes, menqol, postmenopausal syndrome, vasomotor symptoms, vitex extract

## Abstract

Introduction: Postmenopausal women often experience vasomotor and hormonal symptoms due to a decline in estradiol levels, which affects their quality of life (QOL). Botanical extracts like Vitexextract, known for their estrogen- and progesterone-like actions, may offer a safe and effective approach for alleviating postmenopausal symptoms and improving overall quality of life.

Methods: The current study was a prospective, randomized, double-blind, placebo-controlled, monocenter clinical study to evaluate the safety and efficacy of Vitex extract in postmenopausal women aged 40-65 years. Sixty eligible participants were randomized to either the Vitex extract or placebo group equally and instructed to take one capsule daily after breakfast for 60 days. The primary endpoints included changes in the Blatt-Kupperman Index (BKI), Menopause-Specific Quality of Life Questionnaire (MENQOL), Menopause Rating Scale (MRS), and overall QOL scores. The study was prospectively registered with the Clinical Trials Registry of India (CTRI/2025/03/082025).

Results: After 60 days, Vitex extract supplementation led to clinically and statistically significant improvements compared with placebo in all primary outcomes. Notable reductions were observed in BKI, MENQOL, and MRS scores, reflecting better control of vasomotor, psychosocial, physical, and sexual symptoms. Improvements were also seen in the reduction in joint and muscle pain, stress levels, and serum cortisol. Hormonal regulation of luteinizing hormone (LH), follicle-stimulating hormone (FSH), estradiol, and thyroid function, along with reduced rheumatoid factor, indicated anti-inflammatory and endocrine benefits. No adverse effects or lab abnormalities were reported.

Conclusions: In this single-center study, Vitex extract appeared effective and was well tolerated, suggesting its potential as a safe, non-hormonal option for relieving menopausal symptoms and improving quality of life in postmenopausal women. However, these findings should be confirmed in larger, multi-center trials before broader clinical recommendations can be made.

## Introduction

Menopause represents a significant metabolic transition in women, marking the lifelong cessation of menstruation due to a decline in ovarian follicular activity, clinically defined as amenorrhea lasting for 12 consecutive months. This period is frequently accompanied by a complex array of physical, psychological, and emotional symptoms that substantially impact a woman's overall quality of life (QoL) [1].

Globally, menopause and its associated disorders are rising public health concerns, driven by increased life expectancy and a growing aging female population. Projections indicate that by 2030, the number of postmenopausal women will reach 1.2 billion worldwide, with nearly 80% experiencing menopausal symptoms [2]. These symptoms, which can range from mild to severe, include mood swings, depression, sleep disturbances, hot flashes, night sweats, anxiety, and fatigue. Furthermore, long-term health risks such as cardiovascular diseases (CVD), osteoporosis, obesity, diabetes, nonalcoholic fatty liver disease (NAFLD), and dyslipidemia are associated with this transition [3].

The primary physiological disruptions during menopause stem from reduced ovarian secretion of estrogen and progesterone. Estrogen deficiency directly contributes to vasomotor symptoms like hot flashes and night sweats, due to hypothalamic thermoregulatory instability, while alterations in neurotransmitter function are linked to anxiety, irritability, mood disorders, and cognitive changes. Prolonged estrogen loss also increases the risk of dyslipidemia, osteoporosis, and neurodegenerative conditions like Alzheimer's disease [4]. The cumulative effect of these physiological changes extends beyond physical symptoms, adversely affecting social and emotional well-being. Menopausal women often report diminished sleep quality, reduced self-esteem, and decreased work productivity. Consequently, menopausal management strategies must prioritize both symptom reduction and the enhancement of overall QoL [5].

Given the concerns regarding adverse effects associated with long-term hormone replacement therapy (HRT), including thromboembolism, breast cancer, and cardiovascular risks, there has been a growing interest in complementary and alternative medicine (CAM) approaches [6]. Plant-based phytoestrogens are widely regarded as safe and represent a promising approach for the management of menopausal symptoms. These naturally occurring compounds, present in flaxseed, soy, and various traditional medicinal herbs, exhibit structural and functional similarity to 17β-estradiol, allowing them to bind to estrogen receptors and exert estrogenic effects [7].

*Vitex agnus-castus,* commonly known as vitex, chaste tree, or chasteberry, is native to tropical and subtropical regions of Asia. For over 2500 years, it has been extensively used in traditional medicine for managing premenstrual syndrome (PMS), infertility, menstrual irregularities, and menopausal complaints. The fruit of Vitex contains various bioactive constituents, including essential oils, flavonoids, iridoids, and diterpenoids. These compounds are believed to be responsible for the plant's diverse pharmacological activities [8].

Modern pharmacological and clinical studies indicate that the actions of Vitex extract can be explained through multiple interconnected mechanisms. These include dopaminergic pathway modulation, characterized by D₂ receptor activation and subsequent improvement of luteal function, support of the hypothalamic-pituitary-gonadal (HPG) axis through modulation of luteinizing hormone (LH) and follicle-stimulating hormone (FSH) activity, contributing to progesterone production and maintenance of hormonal balance; selective estrogen receptor fine-tuning, particularly through preferential interaction with estrogen receptor beta (ERβ), which may help alleviate estrogen-dominant states, and regulation of neurotransmitter networks involving dopamine, serotonin, and gamma-aminobutyric acid (GABA), thereby promoting mood stability and sleep quality [9-11].

Collectively, these mechanisms provide the physiological basis for the broad spectrum of Vitex extract applications in endocrine-related disorders, including PMS, premenstrual dysphoric disorder (PMDD), mastalgia, menstrual irregularities, luteal-phase dysfunction, infertility associated with mild hyperprolactinemia, perimenopausal and natural menopausal syndromes, and neuroendocrine-related emotional and sleep disturbances [9-11].

In contemporary scientific research, Vitex extract is no longer viewed solely as a phytoestrogen but rather as a multi-target neuroendocrine modulator. Unlike conventional hormone replacement therapy, it does not provide exogenous hormones; instead, it influences the central-pituitary-ovarian axis and neurotransmitter pathways to promote physiological synchronization of endocrine rhythms and maintain hormonal homeostasis. Consequently, Vitex extract has emerged as a representative nutritional endocrine modulator in modern integrative medicine, with the potential to improve endocrine dysfunction associated with stress, sleep disturbances, nutritional inadequacies, and age-related hormonal fluctuations, ultimately contributing to symptom relief and enhanced QoL [9].

The novelty of the present study lies in its comprehensive clinical assessment of Vitex extract, specifically in postmenopausal women, a demographic where robust human trials remain limited. While Vitex extract has been effectively studied for premenstrual and reproductive disorders (PMRD), its targeted role in postmenopausal symptom relief and enhancement of QoL has not been fully established. This study aims to delineate the multidimensional benefits of Vitex extract, including its potential for pain reduction, vasomotor stability, mood regulation, and hormonal balance, through a standardized clinical trial.

## Materials and methods

This was a prospective, randomized, double-blind, placebo-controlled, parallel-group, two-arm study conducted at Sri Venkateswara Hospital, Bengaluru, Karnataka, India, from March 18, 2025, to July 15, 2025, to evaluate the safety and efficacy of Vitex extract in postmenopausal women. Eligible participants were randomized in a 1:1 ratio to receive either Vitex extract or placebo for 60 days. The study protocol and informed consent documents were reviewed and approved by the Sri Venkateshwara Hospital Ethics Committee (protocol number RRS/CL/PM/VITEX/2024, dated October 30, 2024). The study was prospectively registered with the Clinical Trials Registry of India (CTRI/2025/03/082025). Eligible participants were informed about the study objectives, procedures, potential risks, and benefits by the investigator. Written informed consent was obtained from all participants prior to study enrollment in a language understandable to the participant. 

Study participants

Inclusion Criteria

Women aged 40-65 years who were in menopause, defined as the absence of menstruation for 12 consecutive months and having serum FSH levels >30 mIU/mL, were eligible for participation. Participants were required to have a Blatt-Kupperman Index (BKI) score ≥25 or report clinically significant menopausal symptoms despite a BKI score <25.

Exclusion Criteria

Women were excluded if they had used hormone preparations, plant-based hormone substitutes, thyroid hormones, clonidine, anticoagulants, antithrombotic agents, or menopause-related supplements within the past three months, or had a history of drug abuse. Participants receiving osteoporosis treatments such as bisphosphonates, or those with a body mass index (BMI) >30 kg/m², hormone-related cancers, hysterectomy, or severe cardiovascular, thromboembolic, or cerebrovascular diseases were also excluded. Participants with psychiatric disorders, uncontrolled hypertension or diabetes, thyroid dysfunction, abnormal uterine bleeding, significant hepatic or renal impairment (alanine transaminase (ALT)/aspartate aminotransferase (AST) > 3 × upper limit of normal (ULN); estimated glomerular filtration rate (eGFR) < 60 mL/minute/1.73 m²), or endometrial thickness ≥ 7 mm were excluded.

Sample Size Calculation

The sample size was calculated using the formula for comparing two independent means in a superiority trial:: $n=\frac{(Z_{1-\alpha /2}+Z_{1-\beta })^{2}(\sigma _{1}^{2}+\sigma _{2}^{2})}{(\mu _{1}-\mu _{2})^{2}}$, 

where: n = sample size required per group, Z_1−α_ = Z-value corresponding to the two-sided significance level (1.96 for α = 0.05), Z_1−β_ = Z-value corresponding to the desired statistical power (0.84 for 80% power; approximated as 0.85), σ₁ and σ₂ = standard deviations of the two groups, μ₁ and μ₂ = expected mean values of the two groups, and (μ₁ − μ₂) = the expected difference between the group means (effect size).

The sample size estimation was based on the randomized, double-blind, placebo-controlled study by Abbaspoor et al. evaluating Vitex agnus-castus in postmenopausal women [12]. The reported values used for estimation were 4 ± 2.52 for the Vitex group and 6 ± 2.34 for the placebo group. Using a two-sided significance level of 5% (α = 0.05) and 80% statistical power (β = 0.20), the estimated sample size was approximately 23 participants per group. After accounting for an anticipated 10% dropout rate, the sample size was increased to 26 participants per group. To ensure adequate statistical power and to further accommodate potential attrition, the sample size was conservatively rounded up to 30 participants per group, resulting in a total planned sample size of 60 participants (30 participants in each arm).

Disposition of participants

A total of 86 participants were screened, of whom 60 participants were enrolled. Participants who met all eligibility criteria were enrolled and randomized to one of the two treatment groups, with 30 participants in each. A total of 53 participants completed the study, while seven were lost to follow-up (Table 1 and Figure 1).

**Table 1:** Disposition of participants * Participants who completed visit 3 (Day 60)

Particulars	Vitex extract (n=30)	Placebo (n=30)
Number of participants enrolled	30	30
Number of participants completed*	27	26
Number of participants withdrawn	0	0
Lost to follow-up	3	4

**Figure 1:**
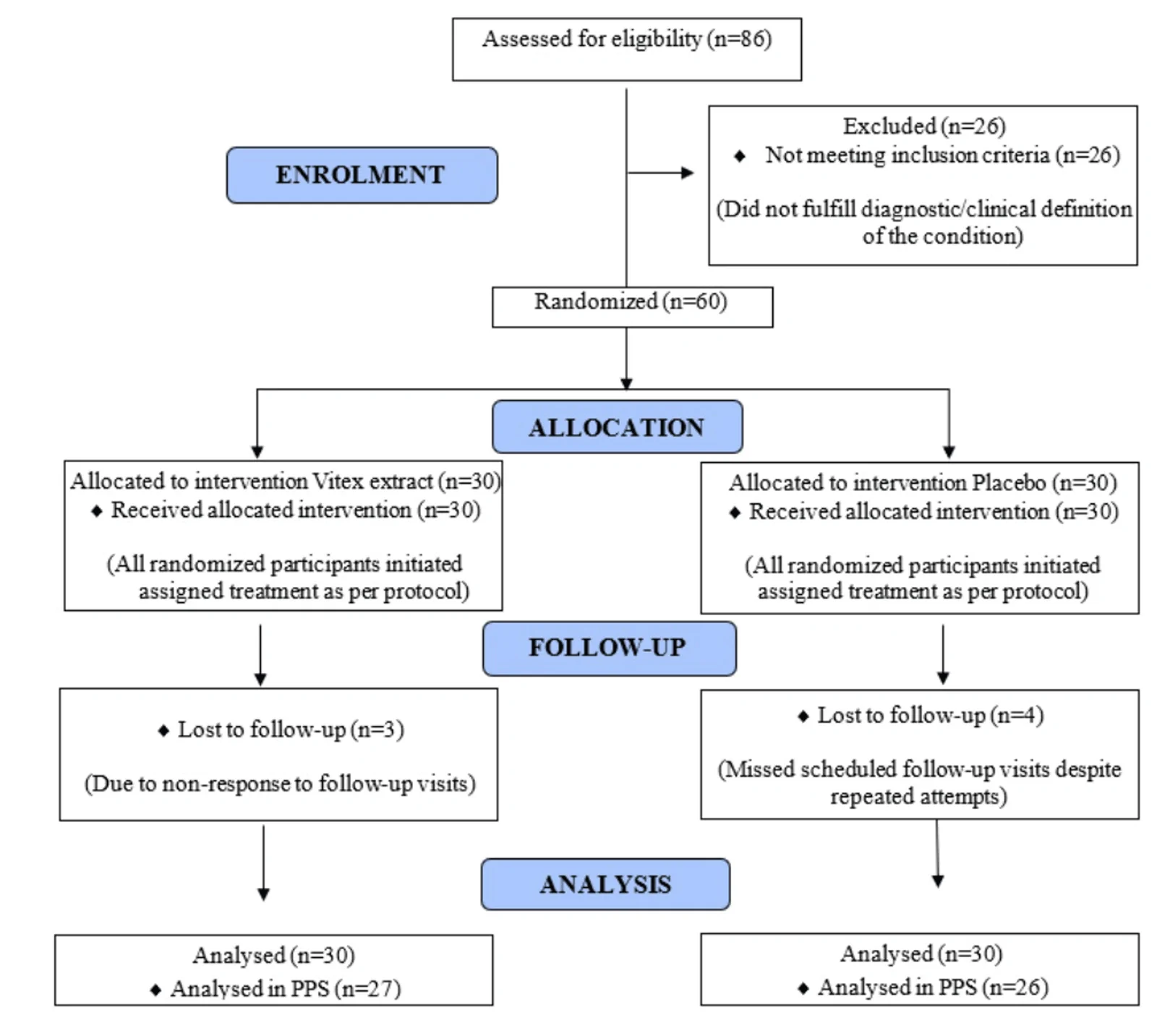
CONSORT diagram showing the flow of participants CONSORT: Consolidated Standards of Reporting Trials

Investigational product (IP)

The IP was a 300 mg capsule containing Vitex extract, standardized to provide 4.5 mg of 100% agnuside, manufactured and registered by Ingex Botanicals Pvt. Ltd., Deganahalli, Karnataka, India. The IP was produced in compliance with Good Manufacturing Practice (GMP) and relevant regulatory standards. Participants in the intervention (Vitex) group received one capsule orally once daily after breakfast for 60 days (two months). The placebo capsules were matched in appearance, size, color, and packaging and were administered according to the same dosing schedule.

Randomization and blinding

The study followed a randomized, double-blind design. Eligible participants were randomly assigned to the different intervention arms according to a computer-generated randomization schedule. Blinded randomization codes were generated by an independent statistician using the R statistical software version 4.4.0 (R Foundation for Statistical Computing, Vienna, Austria), and the active and placebo products were assigned randomly. Block randomization with a fixed block size of 2 was implemented to ensure balanced allocation between the study groups throughout the trial period.

The study was conducted under double-blind conditions, wherein neither the investigators/site personnel nor the participants were aware of the IP allocation. The randomization list was maintained in a password-protected format and was accessible only to designated unblinded pharmacy personnel responsible for IP management and dispensing. The IP was dispensed sequentially according to the predefined randomization schedule as participants qualified for enrollment in the study.

Primary efficacy endpoints

Blatt-Kupperman index (BKI)

Menopausal symptoms were assessed using the BKI, which evaluates 11 climacteric symptoms, including hot flashes, paresthesia, insomnia, nervousness, melancholia, vertigo, weakness/fatigue, arthralgia or myalgia, headache, palpitations, and formication, each rated on a four-point severity scale (0 = none, 1 = mild, 2 = moderate, 3 = severe) [13]. Symptom scores were weighted according to standard BKI criteria (hot flashes ×4; paresthesia, insomnia, and nervousness ×2; all remaining symptoms ×1) and summed to obtain the total BKI score. Total scores were interpreted as ≤15: minimal symptoms, 15-20: mild, 21-35: moderate, and 35+: severe menopausal symptoms (Table 2).

**Table 2:** Kupperman’s Index (BKI) Assessment Method

Symptom	Severity Scale (0–3) *	Weighting Factor	Weighted Score Calculation
Hot flashes (vasomotor)		×4	Severity × 4
Paresthesia (sensory disturbances)	0–3	×2	Severity × 2
Insomnia	0–3	×2	Severity × 2
Nervousness	0–3	×2	Severity × 2
Melancholia (depression/mood)	0–3	×1	Severity × 1
Vertigo	0–3	×1	Severity × 1
Weakness/Fatigue	0–3	×1	Severity × 1
Arthralgia/Myalgia (joint or muscle pain)	0–3	×1	Severity × 1
Headache	0–3	×1	Severity × 1
Palpitations	0–3	×1	Severity × 1
Formication	0–3	×1	Severity × 1

Menopause-Specific Quality of Life Questionnaire (MENQOL)

The MENQOL is a validated instrument used to assess the impact of menopausal symptoms on QoL [14]. It evaluates symptoms across four domains, namely vasomotor, psychosocial, physical, and sexual. Each questionnaire item is scored on a scale from 0 to 6, where 0 indicates absence of symptoms and higher scores indicate greater symptom severity. Domain scores are calculated as the mean of questionnaire items within each domain, with higher scores indicating poorer QoL. The license for the use of the MENQOL was obtained from Mapi Research Trust.

Total Menopause Rating Scale (MRS)

The MRS is a tool used to evaluate the severity of symptoms associated with menopause and their impact on a woman's QoL [15]. It is specifically designed to measure the frequency and intensity of various menopausal symptoms and to assess their effect on daily activities. The MRS score evaluated the severity of menopausal symptoms across somatic, psychological, and urogenital domains, with each item scored on a 0 to 4 scale (0 = no symptoms, 4 = very severe symptoms). Tracking changes in MRS scores provides valuable insights into the overall impact of Vitex extract on menopause-related QoL.

SF-36 (36-Item Short-Form) Health Survey Questionnaire

The SF-36 Health Survey Questionnaire is a widely used QoL assessment tool designed to evaluate an individual’s overall health status and well-being across physical, emotional, and social domains [16]. It measures the impact of health on daily activities, pain, energy levels, emotional health, and general perception of health over the previous four weeks. The scores are interpreted on a scale where higher scores indicate better health-related QoL, improved physical and emotional well-being, and lesser impairment in daily activities, whereas lower scores indicate poorer perceived health status and greater functional limitations.

Biomarker analysis

Biomarker assessments were performed at baseline and Day 60 to evaluate endocrine and stress-related physiological responses associated with the intervention. Changes in biomarker levels over the study period were analyzed to assess the potential effect of the intervention on stress regulation and hypothalamic-pituitary-adrenal (HPA) axis stabilization. Serum concentrations of the hormonal biomarkers, including FSH, LH, cortisol, and estradiol, were assessed to evaluate the endocrine and reproductive hormonal status of postmenopausal women. FSH and LH were measured as indicators of pituitary gonadotropin activity associated with menopausal status; estradiol was evaluated to assess circulating estrogen levels, and cortisol was measured as a marker of physiological stress response.

Quantitative estimation of these biomarkers was performed using commercially available enzyme-linked immunosorbent assay (ELISA) kits according to the manufacturers' instructions (BT Lab, Shanghai Korain Biotech Co., Ltd., Shanghai, China): FSH (Cat. No. E1001Hu), LH (Cat. No. E1037Hu), cortisol (Cat. No. EA0023Hu), and estradiol (Cat. No. EA0030Hu). Blood samples were collected using appropriate vacutainers and centrifuged to obtain serum samples. The serum was subsequently stored at -20°C until further analysis. All biomarker assays were conducted according to validated analytical procedures with appropriate calibration standards and quality control measures in place. The assays were performed in accordance with clinical laboratory standards to ensure accuracy, reliability, and reproducibility of the results.

Statistical analysis

Statistical analyses were performed using IBM SPSS Statistics for Windows, version 25.0 (IBM Corp., Armonk, New York, United States). All statistical tests were conducted at a significance level of 0.05 using two-sided tests unless otherwise specified. Categorical variables were summarized by treatment group using frequency counts and percentages of participants. Continuous variables were summarized using descriptive statistics, including number of participants, mean, standard deviation (SD), median, and range.

The normality of continuous data distributions was assessed using the Shapiro-Wilk test. For inter-group comparisons of continuous variables, an independent two-sample t-test was used when data satisfied the assumption of normality. In cases where normality assumptions were not met, the Wilcoxon rank sum test was applied. For categorical variables, Pearson’s chi-square test or Fisher’s exact test was performed as appropriate based on the distribution of the data. Within-group (intra-group) comparisons between baseline and post-intervention measurements were analyzed using paired t-tests for normally distributed data or Wilcoxon signed rank tests for non-normally distributed data.

Group differences were calculated as the value of the Vitex group minus that of the control group unless otherwise specified. Change from baseline was determined by subtracting the baseline value from the post-intervention value. The baseline value was defined as the last non-missing assessment obtained prior to the first intake of the investigational product for efficacy endpoints and vital signs, or the value measured during the screening visit for hematology and serum biochemistry parameters. All statistical results were reported to two decimal places. p-values were reported to four decimal places, with values less than 0.0001 presented as “<0.0001.” The p-value threshold for statistical significance was set at p < 0.05.

## Results

The baseline (day 0) demographic characteristics of both groups, including age, height, weight, and BMI, are presented in Table 3.

**Table 3:** Demographics of the study participants ^a^ independent t test; ^b^ Mann-Whitney U test

Characteristics	Visits	Vitex extract, mean ± SD	Placebo, mean ± SD	p-value
Age (Years)	Screening	51.15 ± 4.64	51.38 ± 5.56	^a^0.8675
	Mean ± SD			
Height (cm)	Screening	155.93 ± 4.88	157.65 ± 4.71	^a^0.1955
	Mean ± SD			
Weight (Kg)	Screening	61.91 ± 3.90	62.77 ± 4.05	^b^0.4284
	Mean ± SD			
	Visit 3 (Day 60)	61.95 ± 3.90	62.71 ± 4.10	^b^0.4657
	Mean ± SD			
BMI (kg/m²)	Screening	25.50 ± 1.77	25.34 ± 2.44	^a^0.7850
	Mean ± SD			
	Visit 3 (Day 60)	25.52 ± 1.75	25.32 ± 2.45	^a^0.7340
	Mean ± SD			

Primary efficacy outcomes

The primary efficacy outcomes were evaluated to assess the effects of Vitex extract on menopausal symptoms and health-related QoL in postmenopausal women. Efficacy was assessed using validated and widely accepted clinical instruments, including the BKI Total Score [13] for the evaluation of menopausal symptom severity, the MENQOL [14] for assessing menopause-related QoL across vasomotor, psychosocial, physical, and sexual domains, the MRS [15] for measuring the severity of somatic, psychological, and urogenital symptoms, and the QoL questionnaire [16] for evaluating overall well-being and health-related QoL. The primary efficacy outcomes for both the Vitex extract and placebo groups at baseline (Day 0), Visit 2 (Day 30), and Visit 3 (Day 60) are presented in Table 4 and illustrated in Figures 2-5, highlighting the changes observed over the study period and facilitating comparisons between the treatment groups.

**Table 4:** Primary efficacy outcomes ^a^ independent t test; ^b^ Mann-Whitney U test; ^c^ Wilcox signed-rank test **p-value <0.01; ****p-value <0.0001 Min: minimum, Max: maximum

Parameter	Group	Baseline (day 0) (Mean ± SD)	Visit 3 (Day 30) (Mean ± SD)	Visit 3 (Day 60) (Mean ± SD)	% Change Visit 2 (Day 30)	% Change Visit 3 (Day 60)
BKI Total Blatt Kupperman Index Score [12]	Vitex extract	27.26 ± 2.18	24.44 ± 3.52	18.70 ± 3.34	10.34%	31.40%
	Placebo	26.92 ± 1.55	26.27 ± 2.47	25.12 ± 3.64	2.41%	6.69%
	p-value (or)	^a^0.5189	^b^0.1015	^a^<0.0001****	-	-
	% Change					
MENQOL Vasomotor Symptom Score [13]	Vitex extract	3.72 ± 1.06	3.57 ± 0.99	2.89 ± 0.71	–4.03%	–22.31%
	Placebo	3.54 ± 0.80	3.56 ± 0.83	3.38 ± 0.77	0.56%	–4.51%
	p-value (or)	^a^0.4938	^a^0.9880	^a^0.0185*	-	-
	% Change					
Total Psychosocial Symptoms Score	Vitex extract	3.62 ± 0.69	3.49 ± 0.69	2.92 ± 0.62	–3.59%	–1.68%
							Placebo	3.57 ± 0.77	3.51 ± 0.75	3.36 ± 0.77	–19.33%	–5.88%
	p-value (or)	^a^0.7923	^a^0.9253	^a^0.0263*	-	-
	% Change					
	Total Sexual Symptoms Score	Vitex extract	2.96 ± 0.86	2.77 ± 0.98	2.28 ± 0.91	–6.41%	–22.97%
Placebo		3.08 ± 1.01	3.10 ± 1.01	3.03 ± 0.99	0.64%	–1.62%
p-value (or)		^b^0.5595	^b^0.2842	^b^0.0054**	-	-
% Change						
MRS Total Score [14]	Vitex extract	15.74 ± 2.31	14.44 ± 2.36	9.85 ± 2.36	8.25%	37.42%
	Placebo	16.31 ± 2.65	16.19 ± 2.87	15.04 ±3.05	–0.73%	7.79%
	p-value (or)	^b^0.1702	^a^0.0195*	^a^<0.0001****	-	-
	% Change					
QOL [15] Physical Functioning	Vitex extract	394.44 ± 80.06	477.78 ± 81.26	788.89 ±91.29	21.12%	100%
	Placebo	371.15 ± 82.67	378.85 ± 70.96	417.31 ± 111.30	2.07%	12.43%
	p-value (or)	^a^0.3027	^b^<0.0001****	^a^<0.0001****	-	-
	% Change					
Role of limitations due to Physical Health	Vitex extract	133.33 ± 192.15	185.19 ± 179.11	311.11 ± 121.95	38.89%	133%
	Placebo	138.46 ± 194.07	138.46 ± 194.07	161.54 ± 194.07	0	16%
	p-value (or)	^b^0.9309	^b^0.1387	^b^0.0022**	-	-
	% Change					
Role of limitations due to Emotional Problems	Vitex extract	81.48 ± 124.15	129.63 ± 123.46	214.81 ± 109.91	59.09%	163.63%
	Placebo	103.85 ± 121.59	107.69 ± 116.35	111.54 ± 90.89	3.70%	7.40%
	p-value (or)	^b^0.3785	^b^0.5381	^b^0.0007***	-	-
	% Change					
Energy / Fatigue	Vitex extract	215.22 ± 22.29	197.78 ± 23.09	230.37 ± 24.41	–8.10%	7.03%
	Placebo	209.50 ± 28.67	175.38 ± 32.15	168.46 ± 38.02	–16.28%	–19.58%
	p-value (or)	^a^0.4224	^b^0.0071**	^b^<0.0001****	-	-
	% Change					
Emotional Well-Being	Vitex extract	280.74 ± 49.45	304.44 ± 38.56	370.37 ± 30.06	8.44%	31.92%
	Placebo	266.92 ± 42.31	271.54 ± 46.36	276.15 ± 57.42	1.73%	3.45%
	p-value (or)	^a^0.2790	^b^0.0197*	^b^<0.0001****	-	-
	% Change					
Social Functioning	Vitex extract	87.04 ± 32.05	96.30 ± 40.25	101.85 ± 34.62	10.63%	17.01%
	Placebo	92.31 ± 30.63	90.38 ± 34.70	97.12 ± 43.20	–2.09%	5.21%
	p-value (or)	^b^0.5478	^b^0.5693	^b^0.7490	-	-
	% Change					
	Vitex extract	122.78 ± 28.70	134.63 ± 24.45	172.41 ± 16.72	9.65%	40.42%
Pain	Placebo	111.54 ± 31.39	113.46 ± 30.59	116.92 ± 35.36	1.72%	4.82%
	p-value (or)	^a^0.1803	^b^0.0228*	^b^<0.0001****	-	-
	% Change					
General Health	Vitex extract	262.96 ± 38.84	293.52 ± 48.83	397.22 ± 46.68	11.62%	51.05%
	Placebo	274.04 ± 42.12	275.00 ± 44.16	274.04 ± 46.09	0.35%	No change
	p-value (or)	^a^0.3249	^a^0.1534	^a^<0.0001****	-	-
	% Change					

**Figure 2:**
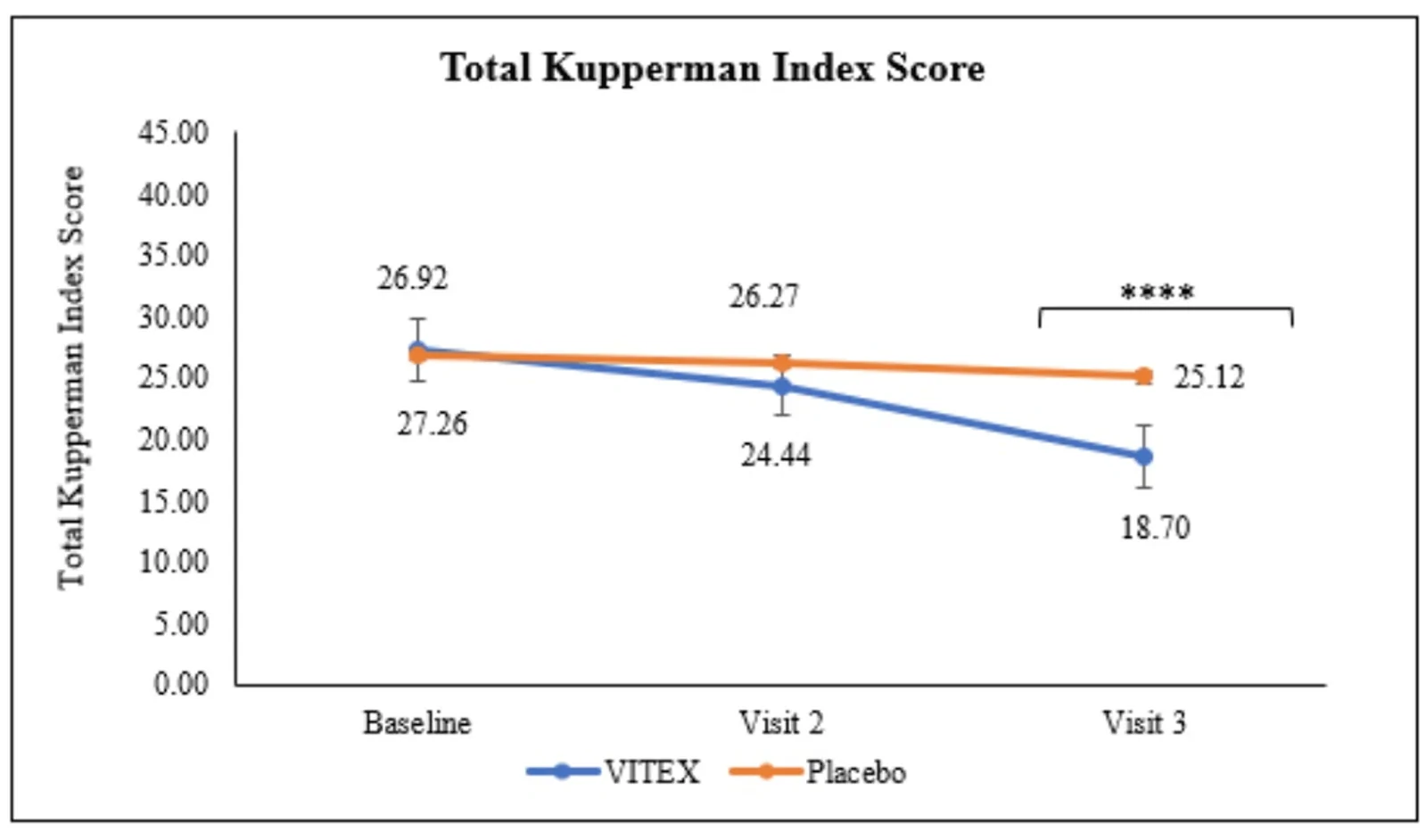
Changes in the Blatt–Kupperman Index (BKI) Total Score. Error bars represent the standard error. ****p-value <0.0001

**Figure 3:**
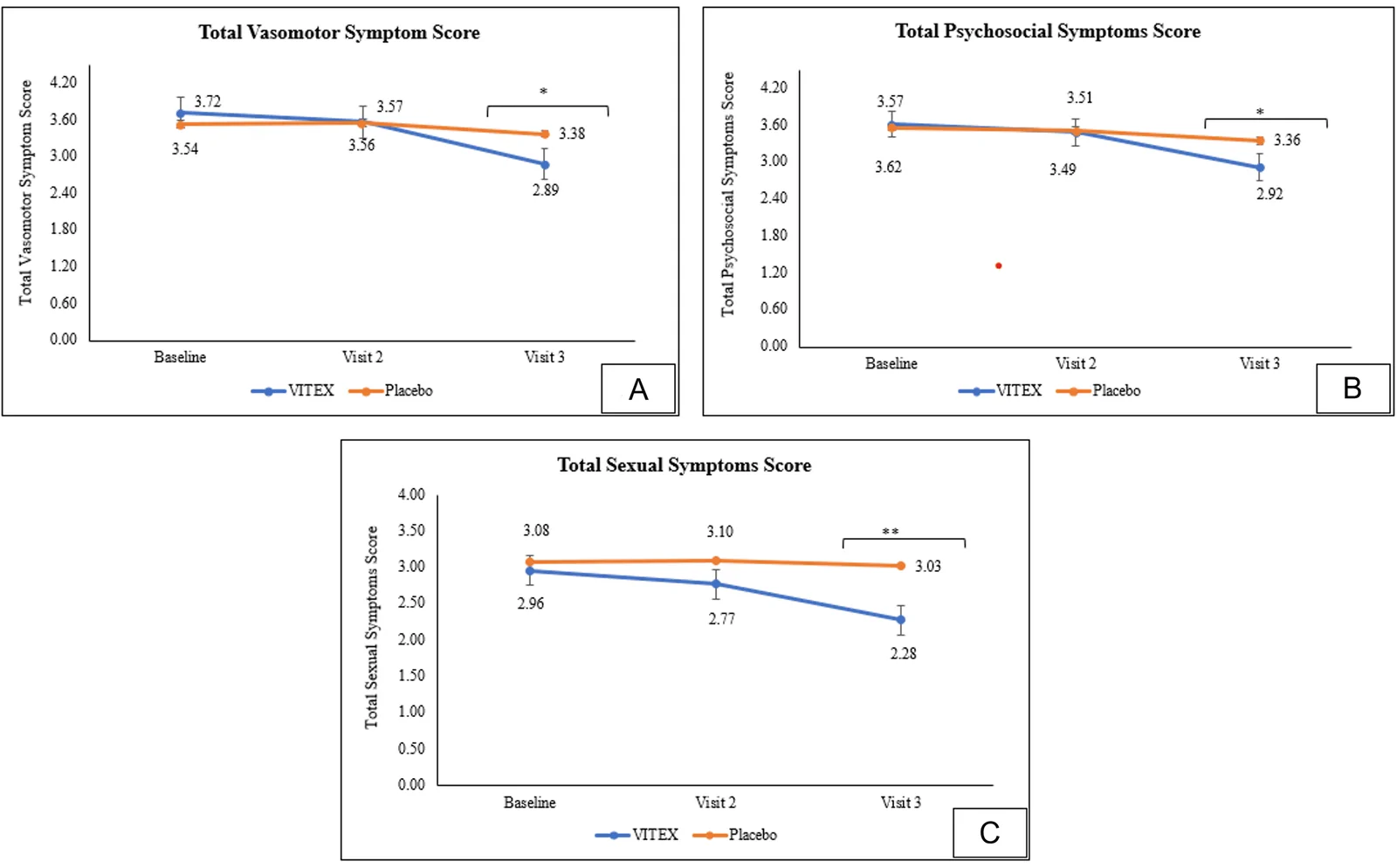
Changes in menopause-specific quality of life questionnaire (MENQOL) (A) Total Vasomotor Symptom Score; (B) Total Psychosocial Symptoms Score; (C) Total Sexual Symptoms Score *p-value < 0.05; **p-value < 0.01

**Figure 4:**
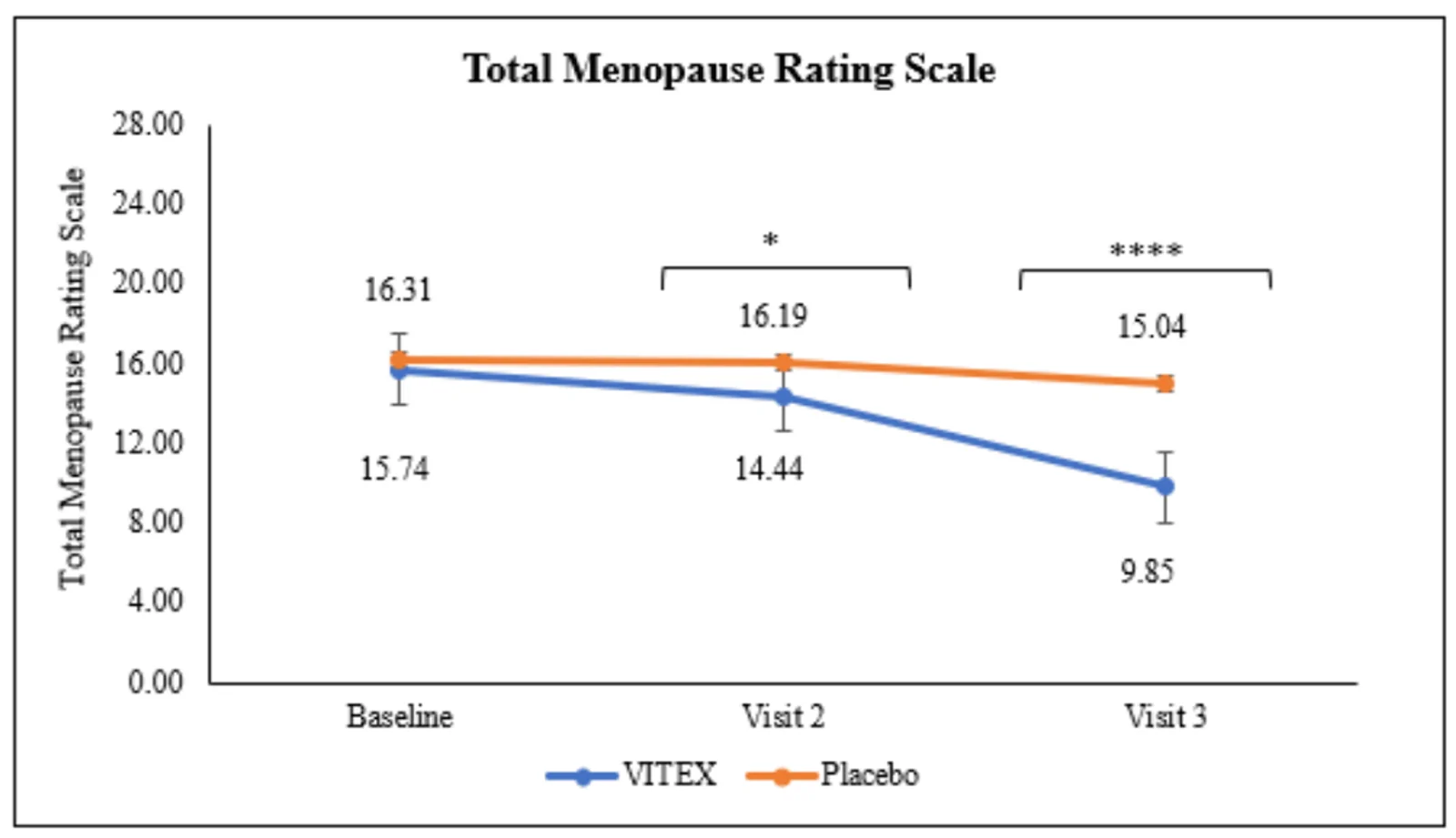
Changes in the total menopause rating scale (MRS) score Error bars represent the standard error. *p-value < 0.05; ****p-value <0.0001

**Figure 5:**
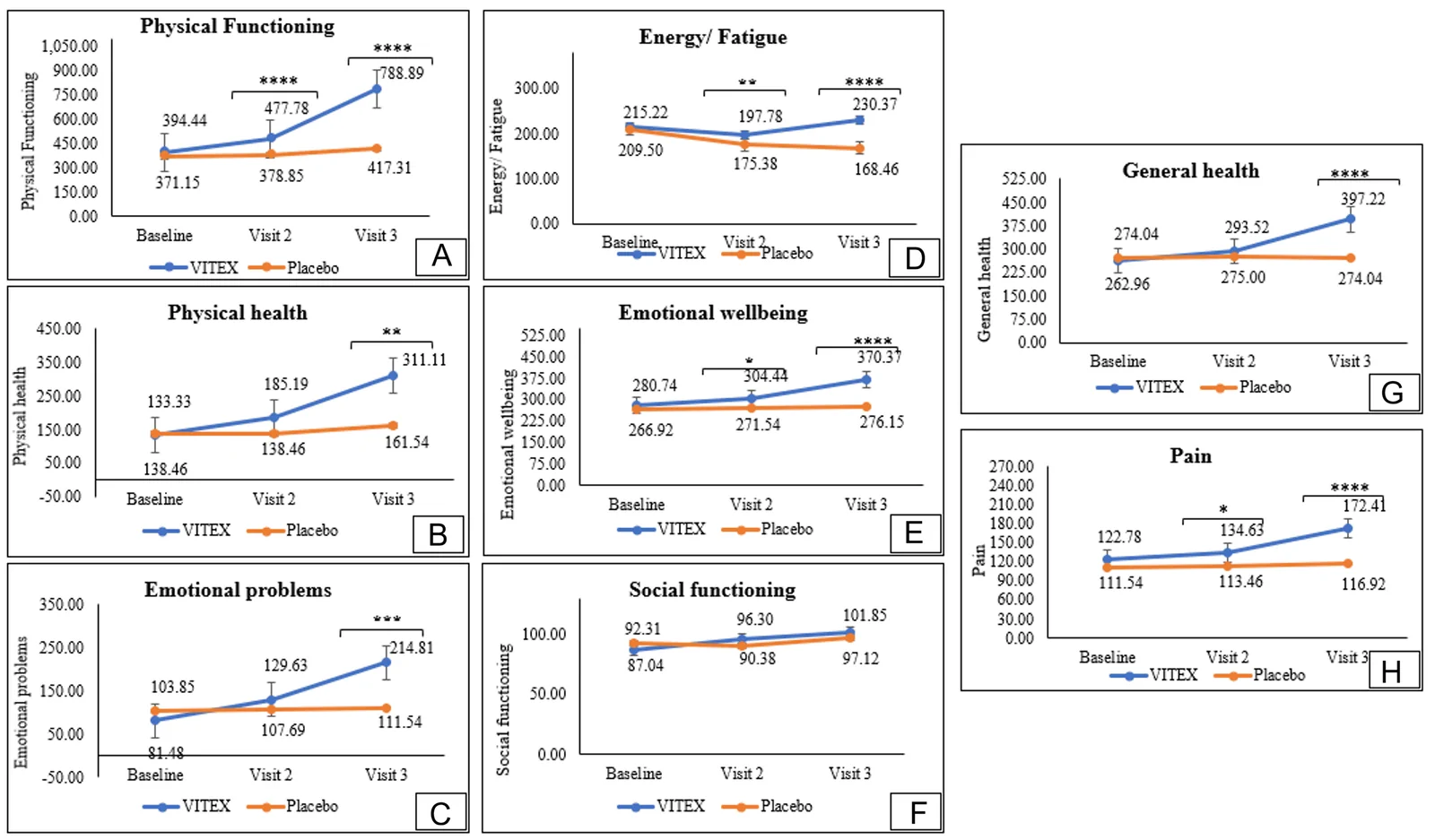
Changes in QoL questionnaire: changes in health-related quality of life assessed using the SF-36 Health Survey (A) Physical functioning; (B) Role limitations due to physical health; (C) Role limitations due to emotional problems; (D) Vitality (energy/fatigue); (E) Emotional well-being (mental health); (F) Social functioning; (G) Bodily pain; (H) General health. Higher score in each domain indicate better health-related quality of life and improvement in the respective domain. Data are presented as mean ± SD. Error bars represent the standard error. Statistical significance is indicated as *p < 0.05; **p< 0.01; ****p-value <0.0001. SF-36: 36-Item Short-Form; QoL: quality of life

The BKI total score significantly decreased from 26.92 at baseline at day 0 to 18.70 at Visit 3 at Day 60 (p < 0.0001) (Figure 2), indicating an improvement in menopausal symptoms. Similarly, the MENQOL questionnaire demonstrated significant improvements in vasomotor, psychosocial, and sexual symptom domains (Figure 3). In addition, the MRS showed a 37.42% reduction in the total score at Visit 3 at Day 60 (p < 0.0001) (Figure 4), confirming the beneficial effect of Vitex extract on menopausal symptom severity. Based on the SF-36 Health Survey graphs (Figure 5A-5H), the Vitex extract group showed a consistent improvement in several domains of health-related QoL compared with the placebo group over the 60-day study period. Since higher SF-36 scores indicate better health status, the observed increases reflect improvements in participants' physical and mental well-being.

Secondary efficacy outcomes

The secondary efficacy outcomes were assessed to further evaluate the effects of Vitex extract on pain perception, vasomotor symptoms, endocrine function, and hormonal status in postmenopausal women. These outcomes included the VAS for pain (mm), which was used to quantify the intensity of pain experienced by participants, and serum cortisol levels, which were measured as an indicator of physiological stress response. Thyroid function was evaluated by measuring T3, T4, and TSH levels. Reproductive hormonal status was assessed through the determination of serum LH, estradiol, and FSH concentrations. In addition, the frequency and severity of hot flushes and night sweats were evaluated as indicators of vasomotor symptoms, while RF was assessed as an additional health-related parameter. The results for VAS, cortisol, T3, T4, TSH, LH, estradiol, and FSH are presented in Table 5; hot flushes and night sweats are presented in Table 6; and rheumatoid factor is presented in Table 7. These parameters were measured at the scheduled study visits to assess changes over time and to compare the effects of Vitex extract with those of placebo.

**Table 5:** Percentage change of secondary efficacy outcomes ^a^ independent t test; ^b^ Mann-Whitney U test **p-value <0.01; ***p-value <0.001 Min: minimum, Max: maximum

Parameter	Vitex group (n=27) (Baseline to Day 60)	Placebo group (n=26) (Baseline to Day 60)	Change from placebo
				VAS Scale for Pain in mm	-45.68	4.69%	-50.37%
Cortisol	-4.39%	-2.20%	-2.19%
Triiodothyronine (T3)	0.30%	-0.15%	0.45%
Thyroxine (T4)	0.80%	-0.24%	1.04%
Thyroid- Stimulating Hormone	-4.05%	0.42%	-4.47%
Luteinizing Hormone (LH)	-4.51%	-0.25%	-4.26%
Estradiol	5.21%	-0.35%	5.56%
Follicle Stimulating Hormone	-6.36%	-0.27%	-6.07%

**Table 6:** Comparison of the two groups as per the degree of hot flush and night sweat on different visit days ^@^ Fisher’s Exact Test **p-value <0.01 ***p-value <0.001

Hot flush and night sweat	Vitex group (n -27)			Placebo group (n=26)			p-value	p-value	p-value
	Baseline (Day 0), n (%)	Visit 2 (Day 30), n (%)	Visit 3 (Day 60), n (%)	Baseline (Day 0), n (%)	Visit 2 (Day 30), n (%)	Visit 3 (Day 60), n (%)	Baseline (Day 0)	Visit 2 (Day 30)	Visit 3 (Day 60)
Not Present	1 (3.7%)	3 (11.1%)	2 (7.4%)	0 (0%)	2 (7.7%)	1 (3.8%)	^@^0.8628	^@^0.0013**	^@^0.0010**
Mild	5 (18.5%)	18 (66.7%)	15 (55.6%)	4 (15.4%)	6 (23.1%)	3 (11.5%)			
Moderate	18 (66.7%)	4 (14.8%)	10 (37%)	20 (76.9%)	16 (61.5%)	20 (76.9%)			
Severe	3 (11.1%)	2 (7.4%)	0 (0%)	2 (7.7%)	2 (7.7%)	2 (7.7%)			

**Table 7:** Comparison of the two groups according to presence of rheumatoid factor on different visit days ^@^ Fisher's Exact Test p<0.05 is statistically significant

Visit	Vutex (n=30)		Placebo (n=30)		p-value
	Positive	Negative	Positive	Negative	
Baseline (Day 0)	2(6.70%)	28(93.30%)	3(10.10%)	27(90.00%)	^@^1
Visit 2 (Day 30)	2(6.70%)	28(93.30%)	3(10.10%)	27(90.00%)	^@^1
Visit 3 (Day 60)	1(3.30%)	29(96.7%)	3(10.10%)	27(90.00%)	^@^0.2373

Severity of PMS

The study included 60 participants, all of whom had moderate BKI scores (20-35) at baseline (day 0). There was no notable change in either group by Visit 2 at Day 30 (Table 8). However, the Vitex extract group started to show improvement at this stage, with 16.67% of participants moving into the mild category, whereas no improvement was seen in the placebo group. By Visit 3 at Day 60, 43.33% of participants in the Vitex extract group had shifted to the mild category, compared with only 10% in the placebo group. This indicates a greater reduction in BKI scores with Vitex extract. Throughout the study, no participants in either group moved into the severe category (Table 8).

**Table 8:** Distribution of the participants of the two groups according to severity of postmenopausal syndrome * p-value <0.05

BKI-Score	Base line (Day 0)		Visit 2 (Day 30)		Visit 3 (Day 60)	
	Vitex group (n=30), n (%)	Placebo group (n=30), n (%)	Vitex group (n=30), n (%)	Placebo group (n=30), n (%)	Vitex group (n=30), n (%)	Placebo group (n=30), n (%)
Mild (15–20)	0 (0%)	0 (0%)	5 (16.67%)	0 (0%)	13 (43.33%*)	3 (10%)
Moderate (20–35)	30 (100%)	30 (100%)	25 (83.33%)	30 (100%)	10 (33.33%*)	23 (76.67%)
Severe (35+)	0 (0%)	0 (0%)	0 (0%)	0 (0%)	0 (0%)	0 (0%)

Adverse events

The adverse events observed in both groups are detailed in Table 9. None of the adverse events were observed as unrelated.

**Table 9:** Adverse events

Adverse Events	Number of Participants		Severity	Relation
	Vitex extract	Placebo		
Respiratory disorders	4	6	Mild	Unrelated
Gastrointestinal disorders	6	5	Mild	Unrelated
Immune system disorders	1	0	Mild	Unrelated
Eye disorders	2	0	Mild	Unrelated

## Discussion

This clinical study was conducted to evaluate the safety and efficacy of Vitex extract in managing symptoms associated with postmenopausal syndrome compared with a placebo. The results clearly showed that Vitex extract significantly improved menopausal symptoms, including vasomotor disturbances, anxiety, and physical discomfort, after 60 days of treatment. On the other hand, the placebo group also showed a mild reduction in symptoms, which may be explained by suggestibility or the placebo effect.

The primary objective of this study was to evaluate the efficacy of Vitex extract in alleviating menopausal symptoms using validated clinical assessment tools. A significant reduction in the BKI total score was observed (Figure 2), decreasing from 26.92 at baseline at Day 0 to 18.70 at Visit 3 at Day 60 (p < 0.0001), indicating a marked improvement in overall menopausal symptoms. Similarly, the MENQOL results (Figure 3) demonstrated significant improvements across the major symptom domains, including vasomotor symptoms, psychosocial symptoms, and sexual symptoms, reflecting an overall enhancement in menopause-related QoL. Furthermore, the MRS results (Figure 4) showed a 37.42% reduction in the total MRS score from baseline at day 0 to Visit 3 at day 60 (p < 0.0001), confirming a statistically significant improvement in the severity of menopausal symptoms following Vitex extract supplementation.

Changes in health-related QoL were assessed using the SF-36 Health Survey, and participants receiving Vitex extract demonstrated significant improvements in multiple SF-36 domains compared with the placebo group by Visit 3. Physical Functioning showed a marked improvement, with scores increasing from 394.44 at baseline at day 0 to 788.89 at Visit 3 at day 60, whereas the placebo group exhibited only a modest increase (371.15 to 417.31). The between-group difference was highly significant (p < 0.0001). Role Limitations due to Physical Health also improved substantially in the Vitex group, increasing from 133.33 to 311.11, compared with a smaller increase in the placebo group (138.46 to 161.54), demonstrating a significant treatment effect (p < 0.01). Similarly, Role Limitations due to Emotional Problems increased from 81.48 to 214.81 in the Vitex group, while the placebo group showed minimal change (103.85 to 111.54), indicating significant improvement (p < 0.001). The Vitality (Energy/Fatigue) domain demonstrated a significant increase in the Vitex group from 215.22 to 230.37, whereas scores in the placebo group progressively declined from 209.50 to 168.46, suggesting improved energy levels and reduced fatigue following supplementation (p < 0.0001).

Emotional Well-being (Mental Health) improved significantly in the Vitex group, with scores increasing from 280.74 to 370.37, compared with only a slight increase in the placebo group (266.92 to 276.15) (p < 0.0001). Although Social Functioning showed a numerical improvement in the Vitex group (92.31 to 101.85), the difference between groups was not statistically significant. The General Health domain demonstrated a significant increase from 274.04 at baseline to 397.22 at Visit 3 in the Vitex group, whereas placebo scores remained essentially unchanged (262.96 to 274.04), indicating a significant enhancement in participants' overall perception of health (p < 0.0001). Finally, the Bodily Pain domain showed significant improvement in the Vitex group, with scores increasing from 122.78 to 172.41, while placebo scores changed minimally (111.54 to 116.92), indicating reduced pain-related limitations (p < 0.0001). Based on the SF-36 Health Survey graphs, the Vitex extract group showed a consistent improvement in several domains of health-related QoL compared with the placebo group over the 60-day study period. Since higher SF-36 scores indicate better health status, the observed increases reflect improvements in participants' physical and mental well-being. Overall, these findings demonstrate that 60 days of Vitex extract supplementation significantly improved both physical and psychological aspects of health-related QoL, with the greatest benefits observed in physical functioning, general health, emotional well-being, vitality, role limitations due to physical and emotional problems, and bodily pain compared with placebo. 

The secondary endpoints were to measure changes in the frequency and severity of vasomotor symptoms like hot flushes and night sweats, musculoskeletal discomfort, and psychological stress, which were assessed through serum cortisol levels. Similar to the present study, earlier randomized, double-blind, placebo-controlled trials have examined the effects of Vitex extract in peri- and post-menopausal women. A study involving 52 women found that eight weeks of Vitex extract led to significant reductions in overall menopausal symptom scores, anxiety, and vasomotor complaints compared with placebo, while no major changes were observed in depression, somatic symptoms, or sexual function [17]. These results are in line with the improvements seen in our study, particularly in emotional well-being, general health, and overall quality-of-life outcomes.

The improvement in the Vitex extract group was much greater compared to placebo, with clinically and statistically significant improvements, confirming the clinical effectiveness of the extract. The findings of this study are consistent with earlier research. A randomized, double-blind study showed Vitex extract significantly reduced total menopausal symptoms [17]. Similarly, a double-blind clinical trial demonstrated that Vitex extract effectively reduced vasomotor symptoms such as hot flushes [12]. Vitex extract was also found to be useful for improving menopausal symptoms [18]. Consistent results from multiple studies provide compelling evidence that Vitex extract possesses potential efficacy in alleviating menopausal symptoms and enhancing QoL.

The mechanism behind these improvements may be related to the hormonal activity of the plant. Vitex contains biologically active compounds, such as flavonoids, which are known to influence the pituitary gland. This gland regulates the secretion of LH, which helps increase progesterone levels and restore hormonal balance. The extract may also help reduce prolactin levels, as shown in earlier animal studies, contributing to the relief of menopausal symptoms and better regulation of hormonal activity [19].

The hormonal findings of this study support these mechanisms, as thyroid hormones (T3, T4, and TSH) remained stable while the Vitex extract group showed a modest but statistically significant decrease in FSH levels (-5.64%) by Visit 3, compared with almost no change in the placebo group. This indicates that Vitex extract may have an effect on gonadotropin regulation, which could be important in managing endocrine changes in postmenopausal women. In addition, the marked reduction in overall symptom scores (approximately 37% decrease in total MRS in the population) is comparable to the improvements reported in earlier clinical trials [20].

Another clinical study reported that Vitex extract significantly reduced both the frequency and intensity of hot flashes (vasomotor symptoms) over an eight-week period compared with placebo, indicating that it may be particularly effective in managing early vasomotor symptoms in postmenopausal women [16]. Beyond the menopausal period, Vitex extract has long been used for various female reproductive conditions such as PMS, luteal phase defects, cyclic breast pain, and other hormone-related disorders. A systematic review of randomized controlled trials found that Vitex extracts were more effective than placebo, pyridoxine, or magnesium in reducing PMS symptoms; in fact, well-standardized Vitex extract preparations were associated with more than twice the likelihood of symptom improvement compared with placebo [21,11].

Pharmacological studies showed that Vitex extract contains a diverse range of phytochemicals-such as flavonoids, iridoids, diterpenes, phenolic acids, and terpenes-that may produce estrogen-like, antioxidant, dopaminergic, and neuroendocrine effects. The dopaminergic actions of Vitex extract, in particular, may influence the pituitary gland’s release of prolactin, LH, and FSH, which can indirectly affect ovarian steroid production and overall hormonal regulation [22]. This type of hormonal modulation may help improve well-being, increase energy levels, and reduce menopausal symptoms over time. The combined phytoestrogenic, dopaminergic, antioxidant, and possible neuroendocrine actions of Vitex extract give it a broad and multi-targeted therapeutic profile. With increasing interest in herbal and phytoestrogen-based treatments, the findings of this study support the idea that Vitex can enhance several aspects of postmenopausal health and QoL without causing major hormonal disturbances.

Although previous reviews show that Vitex extract is usually well tolerated and side effects are uncommon, some reactions such as digestive discomfort, headaches, or mild hormonal shifts can occur, and its long-term safety in postmenopausal women still needs more investigation [23]. In this study, Vitex extract use in postmenopausal participants produced notable and clinically meaningful improvements in menopausal symptoms, overall QoL, emotional well-being, pain, energy, social functioning, and general health. The hormonal assessments showed modest but favorable changes, including a reduction in FSH and stable thyroid hormone levels, suggesting that Vitex extract may act as a phytoestrogenic and neuroendocrine modulator rather than a direct hormone replacement. These outcomes are consistent with earlier clinical trials and pharmacological studies indicating that Vitex extract can reduce menopausal symptoms, especially vasomotor complaints, anxiety, and overall symptom burden. While these findings are promising, larger and more robust studies with standardized extracts and long-term follow-up are required to confirm the effectiveness and safety of Vitex extract as a treatment option for postmenopausal syndrome.

This study has several limitations that should be considered when interpreting the findings. The intervention period was limited to 60 days, which may not fully capture the long-term efficacy and safety profile of Vitex extract in postmenopausal women. In addition, the relatively modest sample size and single-center study design may restrict the generalizability of the results to broader and more diverse populations. The study also did not assess variations in treatment response based on menopausal stage, baseline hormonal status, or lifestyle-related factors that could influence outcomes. Furthermore, the absence of extended follow-up after completion of supplementation limits conclusions regarding the sustainability of symptom improvement and long-term hormonal stability, highlighting the need for larger multicenter trials with longer intervention durations.

## Conclusions

This clinical study demonstrates that Vitex extract, administered for 60 days, provides meaningful clinical benefits for postmenopausal women by improving menopausal symptoms and overall quality of life more effectively than placebo. Participants receiving Vitex extract experienced notable improvements across multiple domains, including vasomotor symptoms, psychological well-being, musculoskeletal discomfort, physical functioning, social and emotional health, and general quality of life, with benefits emerging early and strengthening over the study period. These symptomatic improvements were supported by favorable hormonal trends, such as healthier gonadotropin regulation and mild enhancement of estradiol levels, along with reduced cortisol values indicating better stress modulation and emotional stability.

The treatment was well tolerated, with vital signs, physical examinations, and safety assessments showing no clinically significant abnormalities, confirming a strong safety profile for short-term use. Together, these results suggest that Vitex extract may act through both hormonal and neuroendocrine pathways and may serve as a safe, natural, non-hormonal alternative for managing postmenopausal symptoms. However, the study also presents certain limitations, including a relatively short treatment duration, a modest sample size, and the absence of long-term follow-up to assess sustained efficacy, safety, and hormonal stability. Additionally, the study did not evaluate variations in response based on menopausal stage, lifestyle factors, or baseline hormone levels, which may further influence clinical outcomes. Considering these gaps, future research should involve large-scale sample sizes, multicenter trials with longer treatment periods and extended follow-up to validate the therapeutic potential of Vitex and clarify its broader role in supporting women’s health during menopause.
